# RETRACTION: Downregulation of Long Noncoding RNA LUCAT1 Suppresses the Migration and Invasion of Bladder Cancer by Targeting miR‐181c‐5p

**DOI:** 10.1155/bmri/9842795

**Published:** 2025-10-18

**Authors:** BioMed Research International

RETRACTION: Y. Chen, W. Zhang, L. Shen, et al., “Downregulation of Long Noncoding RNA LUCAT1 Suppresses the Migration and Invasion of Bladder Cancer by Targeting miR‐181c‐5p,” *BioMed Research International* 2020 (2020): 4817608, https://doi.org/10.1155/2020/4817608.

The above article, published online on 17 November 2020 in Wiley Online Library (wileyonlinelibrary.com), has been retracted by John Wiley & Sons Ltd.

The retraction has been agreed following an investigation into concerns originally flagged by *Hoya camphorifolia* on PubPeer [1], which identified multiple instances of image duplication.

Following these concerns being raised, the authors contacted the publisher to state that this was due to the results being obtained by a third‐party company. The retraction of the article was agreed due to concerns around the validity of results.
